# Application and prognostic analysis of endoscopic sinus surgery combined with multidisciplinary team management in rhino-orbito-cerebral mucormycosis

**DOI:** 10.3389/fsurg.2026.1854275

**Published:** 2026-08-05

**Authors:** Tingting Liu, Qiuliang Zhao

**Affiliations:** Department of Otolaryngology, Head and Neck Surgery, Central Hospital Affiliated of Shandong First Medical University (Jinan Central Hospital), Jinan, China

**Keywords:** antifungal therapy, endoscopic sinus surgery, multidisciplinary team, prognosis, rhino-orbito-cerebral mucormycosis

## Abstract

**Objective:**

To evaluate the clinical value of endoscopic sinus surgery (ESS) combined with a multidisciplinary team (MDT) approach in rhino-orbito-cerebral mucormycosis (ROCM) and identify independent prognostic factors.

**Methods:**

This retrospective cohort study enrolled 22 consecutive patients with ROCM managed by a standardized MDT protocol between January 2020 and June 2024.Clinical data covering endoscopic surgical strategies and cross-specialty MDT collaboration were systematically extracted. Univariate chi-square analysis and multivariate binary logistic regression were performed to screen mortality predictors. Kaplan–Meier survival curves with log-rank tests were generated for survival comparisons.

**Results:**

The cohort included 14 males and 8 females with a mean age of 58.6 ± 10.3 years. Diabetes mellitus was the dominant underlying comorbidity (18/22, 81.8%), among whom six patients presented with diabetic ketoacidosis (27.3%). 18 patients (81.8%) received endoscopic debridement, and 10 of these surgical patients (55.6%) underwent concurrent endoscopic optic nerve decompression. Histopathology confirmed characteristic broad, aseptate, right-angle branching hyphae; Rhizopus species were isolated from 6 patients via fungal culture and metagenomic next-generation sequencing (mNGS). At the predefined 6-month primary follow-up endpoint, 12 patients (54.5%) met composite remission criteria, while 10 patients (45.5%) died of ROCM-related complications. Multivariate logistic regression identified intracranial extension as the sole independent risk factor for mortality (OR = 28.5, 95% CI: 2.1–387.4, *P* = 0.011). Early surgery performed within 72 h of symptom onset showed a trend toward reduced mortality (OR = 0.18, 95% CI: 0.02–1.52, *P* = 0.11), and well-controlled glycemia (HbA1c ≤ 7.0%) exhibited a protective tendency (OR = 0.25, 95% CI: 0.03–2.08, *P* = 0.20), yet neither variable reached statistical significance after multivariate adjustment. Kaplan–Meier survival analysis revealed significantly longer survival among patients without intracranial fungal invasion (log-rank *P* < 0.001).

**Conclusion:**

Endoscopic sinus surgery serves as the core intervention to eradicate primary sinonasal lesions in ROCM. Structured MDT collaboration optimizes surgical timing and standardized comorbidity management. Early precise endoscopic debridement combined with standardized long-term antifungal therapy substantially improves clinical outcomes. Timely endoscopic debridement within 72 h and strict glycemic control represent critical modifiable factors to reduce mortality risk.

## 1 Introduction

Mucormycosis is a severe opportunistic invasive fungal disease, mainly caused by fungi of the order Mucorales, including Rhizopus and Mucor species (1). The global incidence of mucormycosis has risen significantly, particularly following the COVID-19 pandemic. India reported over 49,000 cases of acute invasive fungal rhinosinusitis (AIFR) within three months during the second wave of COVID-19 in 2021 (2), while European and North American centers documented a 2- to 3-fold increase in mucormycosis cases among immunocompromised populations (3, 4). The estimated global burden is approximately 0.5–1.0 cases per 100,000 population annually (5).

Clinical manifestations of mucormycosis are classified into six major forms: rhino-orbito-cerebral (ROCM), pulmonary, cutaneous, gastrointestinal, disseminated, and miscellaneous (including renal and isolated cerebral involvement) (6). Among these, ROCM is the most common presentation, accounting for approximately 40–50% of all mucormycosis cases, and carries the highest mortality rate, approaching 80% in advanced disease (7). The pathophysiology is characterized by angioinvasion, tissue necrosis, and rapid dissemination along vascular channels.

Principal risk factors include uncontrolled diabetes mellitus (particularly diabetic ketoacidosis), hematologic malignancies, solid organ transplantation, deferoxamine therapy, and corticosteroid use (8). Diabetes mellitus represents the predominant risk factor globally, present in 54–76% of ROCM cases, as hyperglycemia induces ketoacidosis and increases free iron availability—both critical growth factors for Mucorales (9).

ROCM often presents with a “symptom dissociation” —patients frequently present with headache, visual disturbance, or altered mental status rather than nasal symptoms, leading to delayed diagnosis in over 50% of cases (10).

This diagnostic challenge necessitates multidisciplinary collaboration, as single-specialty management is insufficient to address sinonasal debridement, orbital intervention, intracranial complications, metabolic control, and antifungal therapy simultaneously (11).

As a core discipline in the management of ROCM, otolaryngology plays a critical role in eradicating primary sinus lesions via endoscopic sinus surgery (ESS) (12), facilitating multidisciplinary collaboration, and managing the postoperative sinus cavity. ESS, with its minimally invasive nature and excellent visualization, has become the preferred surgical approach for removing sinus lesions. Nevertheless, the optimal timing of surgery and the standardization of multidisciplinary collaboration still require further investigation. This study presents a 4-year, single-center experience of 22 consecutive ROCM patients managed through a structured MDT protocol, with emphasis on surgical timing, technical nuances, and prognostic determinants.

## 2 Materials and methods

### 2.1 Ethics approval and study design

This retrospective cohort study was conducted at a tertiary academic medical center, from January 2020 to June 2024. The study was approved by the Institutional Ethics Committee (Approval No. 20260702004). Written informed consent was obtained from all patients or their legal guardians. The study adheres to the STROBE guidelines for observational studies (13).

### 2.2 Patient selection

A total of 22 patients with ROCM treated by our MDT team from January 2020 to June 2024 were included in this study. **Inclusion criteria:** (1) Age ≥18 years; (2) Clinical diagnosis of ROCM based on host factors (immunocompromised state, particularly diabetes mellitus or diabetic ketoacidosis), clinical manifestations (nasal, orbital, or cerebral symptoms), and radiological evidence of sinus involvement with bone destruction; (3) Histopathological or microbiological confirmation of Mucorales; (4) Complete medical records and ≥6-month follow-up data available. **Exclusion criteria:** (1) Isolated rhinosinusitis without orbital or cerebral extension; (2) Other fungal infections (e.g., aspergillosis) confirmed by culture or histopathology; (3) Incomplete clinical data or loss to follow-up within 6 months; (4) Patients who declined MDT evaluation or standard treatment protocols.

### 2.3 Diagnostic criteria

The diagnosis of mucormycosis followed the 2019 European Confederation of Medical Mycology (ECMM) global guidelines (4): (1) Presence of underlying immunocompromising conditions; (2) Clinical manifestations of rhino-orbital-cerebral involvement or imaging findings showing sinus lesions with bone destruction; (3) Histopathological or microbiological detection of Mucorales hyphae.

### 2.4 Standardized imaging protocol

All patients underwent high-resolution computed tomography (HRCT) of the paranasal sinuses (slice thickness ≤1 mm) and contrast-enhanced magnetic resonance imaging (MRI) of the brain and orbits. CT evaluation focused on bone destruction patterns, soft tissue involvement, and orbital wall integrity. MRI is the preferred imaging modality when ROCM is suspected and surgical biopsy is planned. Given that ROCM is characterized by progressive tissue infiltration and necrosis, a focal absence of contrast enhancement within an otherwise homogeneous sinus mucosa confirms the presence of a necrotic area. This radiological feature is commonly referred to in the literature as the “black turbinate sign”, which serves as an early sentinel sign (14). Initial MRI examination has shown that up to 83% of patients with ROCM have involvement of the extraconal fat, intraconal fat, extraocular muscles, or optic nerve. Epidural, intracerebral, and cavernous sinus involvement are seen in 5% to 10% of patients, respectively (15).

### 2.5 Multidisciplinary team (MDT) standardized workflow

The MDT comprised otorhinolaryngology (lead coordinator), neurosurgery, infectious diseases, endocrinology, ophthalmology, anesthesiology, and intensive care medicine. The standardized workflow included emergency assessment (0–2 hours), MDT activation (2–4 hours), metabolic optimization and imaging (4–12 hours), surgical intervention (≤72 hours), ICU management (7–14 days), and endoscopic surveillance (8–12 weeks).

### 2.6 Surgical technique

All endoscopic procedures were performed under general anesthesia by a senior otorhinolaryngologist (≥10 years endoscopic skull base experience) with image guidance (Medtronic StealthStation S7).

#### 2.6.1 Sinonasal debridement

The surgical principle was “precise debridement with functional preservation.” The operative sequence included: (1) Bilateral sphenoidotomy with posterior septectomy for bilateral access; The sphenoid ostium was enlarged to 1.0 cm × 1.2 cm to ensure complete drainage. (2) Extended ethmoidectomy with removal of necrotic tissue; (3) Maxillary antrostomy via the natural ostium, enlarged to ≥1.0 cm; (4) Frontal sinusotomy via the frontal recess; (5) Targeted debridement of necrotic mucosa at the interface of blackened and normal-appearing tissue, where cotton-like fungal exudate was typically identified. Tissue specimens were obtained from this junction for histopathology and culture.

#### 2.6.2 Endoscopic optic nerve decompression

Surgical indications for optic nerve decompression: (1) Visual acuity ≤0.3 or progressive visual loss; (2) Radiological evidence of optic nerve edema (T2-FLAIR hyperintensity) or orbital apex syndrome; (3) Intraoperative visualization of optic nerve compression by necrotic tissue or fungal mass. The technique involved: (1) Image-guided identification of the optic canal; (2) High-speed diamond burr (Medtronic XPS 3000) removal of the medial orbital wall and optic canal medial wall (length 1.5–2.0 cm); (3) Longitudinal incision of the optic nerve sheath with microscissors; (4) Confirmation of cerebrospinal fluid egress or sheath decompression.

#### 2.6.3 Intraoperative irrigation

The surgical cavity was irrigated with 500 mL of amphotericin B solution (5 mg/100 mL normal saline). Hemostasis was achieved with gelatin sponge packing, removed 48 hours postoperatively.

### 2.7 Postoperative standardized management

#### 2.7.1 Systemic antifungal therapy

All patients received liposomal amphotericin B (AmBisome, Gilead Sciences) intravenously, initiated at 5 mg/kg/day and titrated to 10 mg/kg/day based on renal function, for 4–6 weeks (16). Patients with creatinine elevation >133 *μ*mol/L (*n* = 4) were switched to flucytosine (100 mg/kg/day, divided q6 h) plus posaconazole (400 mg bid). Step-down to oral posaconazole (400 mg bid) was administered for ≥6 months total duration.

#### 2.7.2 ICU management

Sixteen patients (72.7%) were transferred to the ICU postoperatively for respiratory support, immunomodulation (intravenous immunoglobulin 20 g/day × 3 days), and nutritional support. Mean ICU stay was 7.5 ± 2.3 days.

#### 2.7.3 Endoscopic surveillance

Twice-weekly endoscopic debridement was performed to remove crusts and granulation tissue, with amphotericin B saline irrigation, until complete mucosal epithelialization (mean 8–12 weeks).

### 2.8 Microbiological diagnosis

Tissue specimens obtained during endoscopic surgery were subjected to: (1) Direct microscopy with 10% potassium hydroxide (KOH) preparation and calcofluor white staining; (2) Histopathological examination with hematoxylin-eosin (HE) and Grocott’s methenamine silver (GMS) staining; (3) Fungal culture on Sabouraud dextrose agar (SDA; Becton Dickinson, Franklin Lakes, NJ, USA) with chloramphenicol and gentamicin, incubated at 25°C and 37°C for 7–14 days; Species identification from culture was performed using polymerase chain reaction (PCR) amplification and sequencing of the internal transcribed spacer (ITS) region, with results reported as Rhizopus spp; (4) Matrix-assisted laser desorption/ionization time-of-flight mass spectrometry (MALDI-TOF MS) for species identification; (5) Metagenomic next-generation sequencing (mNGS) of tissue, cerebrospinal fluid, or abscess aspirate using the IDseq platform (Jinan KingMed Diagnostics Laboratory, Jinan, China) for rapid pathogen detection, with species-level identification based on unique mapped reads to reference genomes.

### 2.9 Standardized outcome definitions

**Clinical remission:** Complete resolution of clinical symptoms (headache, visual disturbance, facial pain), radiological evidence of lesion clearance with mucosal epithelialization on CT/MRI, and negative fungal culture from nasal secretions.
**Death:** Mortality attributable to ROCM-related complications (intracranial infection progression, multiple organ failure, or fatal hemorrhage).
**Visual outcome:** Snellen chart visual acuity testing performed at baseline, postoperative day 7, postoperative month 1, month 3, and month 6 for standardized longitudinal visual function recording.

### 2.10 Statistical analysis

Statistical analysis was performed using SPSS 26.0 (IBM Corp., Armonk, NY, USA). Continuous variables were expressed as mean ± standard deviation (SD) or median (interquartile range); categorical variables as frequencies and percentages. Group comparisons were performed using chi-square test or Fisher's exact test for categorical variables, and independent t-test or Mann–Whitney U test for continuous variables.

Kaplan–Meier curves were constructed for time-to-death and time-to-remission, with log-rank test for between-group comparisons. Binary logistic regression was performed with mortality as the dependent variable. Independent variables included age (continuous), diabetes control (HbA1c ≤ 7.0% vs >7.0%), surgical timing (≤72 hours vs >72 hours), intracranial extension (present vs absent), and renal function (eGFR ≥60 vs <60 mL/min/1.73m^2^). Variables with *P* < 0.10 in univariate analysis were entered into the multivariate model. Odds ratios (OR) with 95% confidence intervals (CI) were calculated. A two-tailed *P* < 0.05 was considered statistically significant. Given the small sample size (*n* = 22, 10 events), the minimum detectable OR for a binary predictor with 80% power and *α*=0.05 is approximately 8.5.

## 3 Results

### 3.1 Clinical and sociodemographic characteristics

The cohort comprised 22 patients (14 males, 8 females), aged 45–76 years (mean 58.6 ± 10.3 years). Disease duration was 3–12 days (mean 7.2 ± 2.5 days). Underlying diseases included diabetes mellitus in 18 (81.8%, including 6 with diabetic ketoacidosis), hypertension in 12 (54.5%), chronic kidney disease in 4 (18.2%), and no underlying disease in 2 (9.1%). Presenting symptoms included sudden headache (12, 54.5%), unilateral vision loss (10, 45.5%), nasal obstruction (8, 36.4%), nasal black eschar (6, 27.3%), facial pain/swelling (5, 22.7%), altered consciousness (4, 18.2%), and fever (3, 13.6%). Baseline characteristics are summarized in Table 1.

**Table 1:** Baseline clinical and sociodemographic characteristics of 22 ROCM patients.

Characteristic	*n* (%) or Mean ± SD
Age, years	58.6 ± 10.3
Male sex	14 (63.6)
Diabetes mellitus	18 (81.8)
— With diabetic ketoacidosis	6 (27.3)
Hypertension	12 (54.5)
Chronic kidney disease	4 (18.2)
No underlying disease	2 (9.1)
Initial presentation to Neurosurgery	14 (63.6)
Initial presentation to Otorhinolaryngology	8 (36.4)
Symptom duration, days	7.2 ± 2.5
Baseline HbA1c, % (diabetic patients)	10.8 ± 2.4
Baseline creatinine, μmol/L	142.3 ± 58.7

### 3.2 Imaging findings

**Figure 1:**
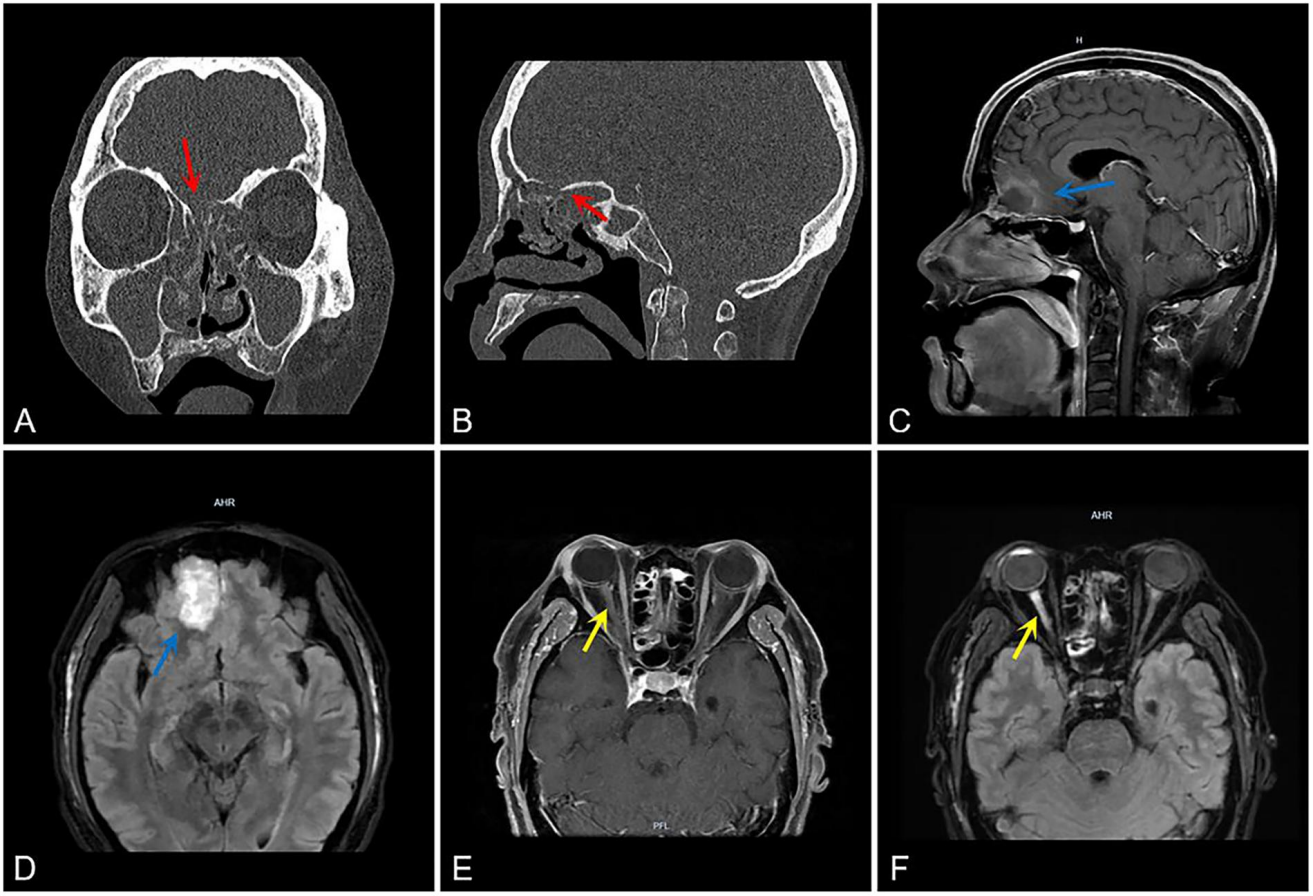
Imaging findings. Sinus CT shows sinus mucosal thickening with associated soft tissue density, along with bony destruction of the left orbital wall and skull base **(A,B)** (red arrows). Cranial MRI reveals intracranial abscess formation **(C,D)** (blue arrows). Cranial MRI demonstrates irregular soft tissue opacity in the right ethmoid sinus and optic nerve edema (T2-Flair hyperintensity) with mild enhancement **(E,F)** (yellow arrows).

**CT findings:** Unilateral sinus involvement in 20 (90.9%), bilateral in 2 (9.1%). Bone destruction was present in 16 (72.7%): orbital wall invasion in 10 (45.5%) and skull base erosion in 6 (27.3%). Typical findings included irregular soft tissue opacification in the ethmoid sinuses with orbital wall bone absorption and blurring of the orbital apex fat plane (Figures 1A,B).
**MRI findings:** The “black turbinate sign” was observed in 15 (68.2%). Extraocular muscle involvement in 8 (36.4%), optic nerve thickening in 10 (45.5%), and optic nerve T2-FLAIR hyperintensity in 4 (18.2%). Intracranial extension was identified in 6 (27.3%): intracranial abscess in 2 (9.1%) and cavernous sinus involvement in 4 (18.2%) (Figures 1C–F).

### 3.3 Treatment and surgical outcomes

**Figure 2:**
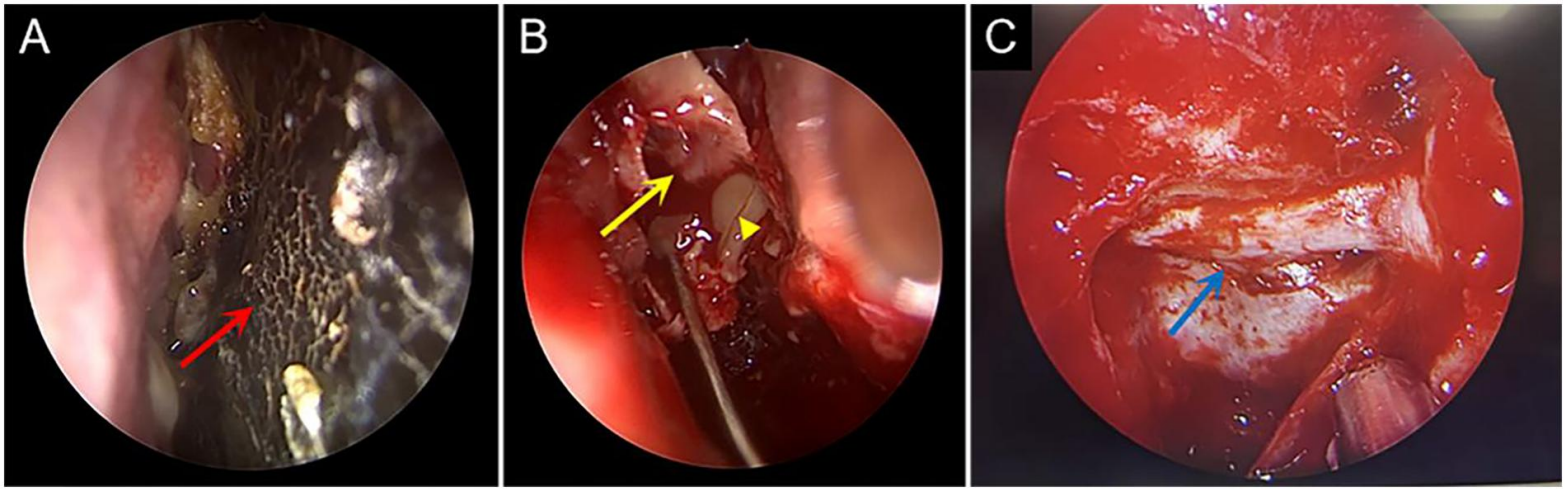
Representative intraoperative findings. **(A)** Black eschar on the lateral wall of the left nasal cavity (red arrow). **(B)** Cotton-like material and purulent secretions in the ethmoid sinus, with bone destruction (yellow arrow). **(C)** Endoscopic optic nerve decompression (blue arrow).

Eighteen patients (81.8%) underwent definitive endoscopic sinus debridement, while four patients declined surgical intervention due to severe pre-operative multiple organ failure, family refusal based on perceived high surgical risk, and terminal pre-operative intracranial hemorrhage. Representative intraoperative findings are shown in Figure 2.

### 3.4 Microbiological diagnosis

**Figure 3:**
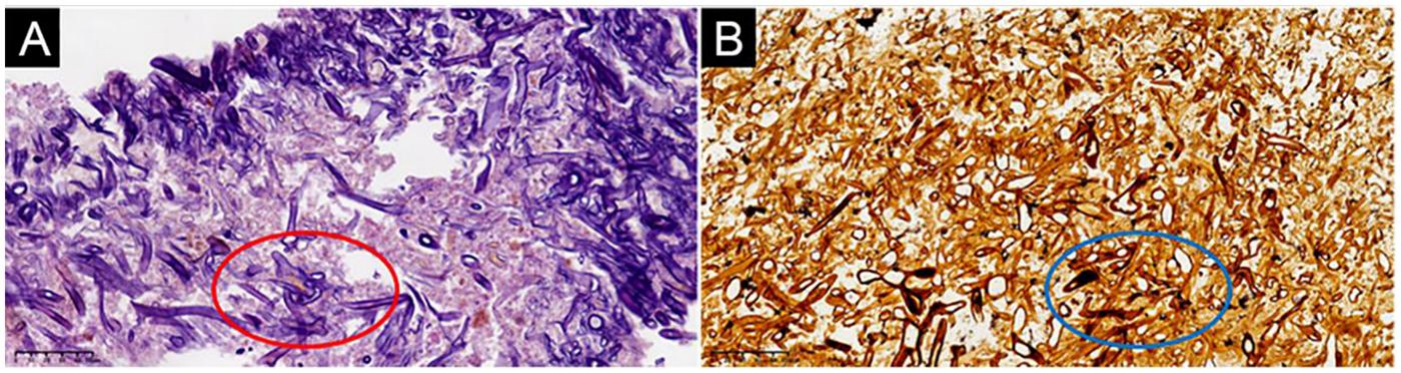
Histopathological findings in rOCM. **(A)** Hematoxylin-eosin staining showing broad, ribbon-like, aseptate hyphae with right-angle branching (×400). **(B)** Grocott's methenamine silver staining highlighting fungal elements in necrotic tissue (×400).

**Figure 4:**
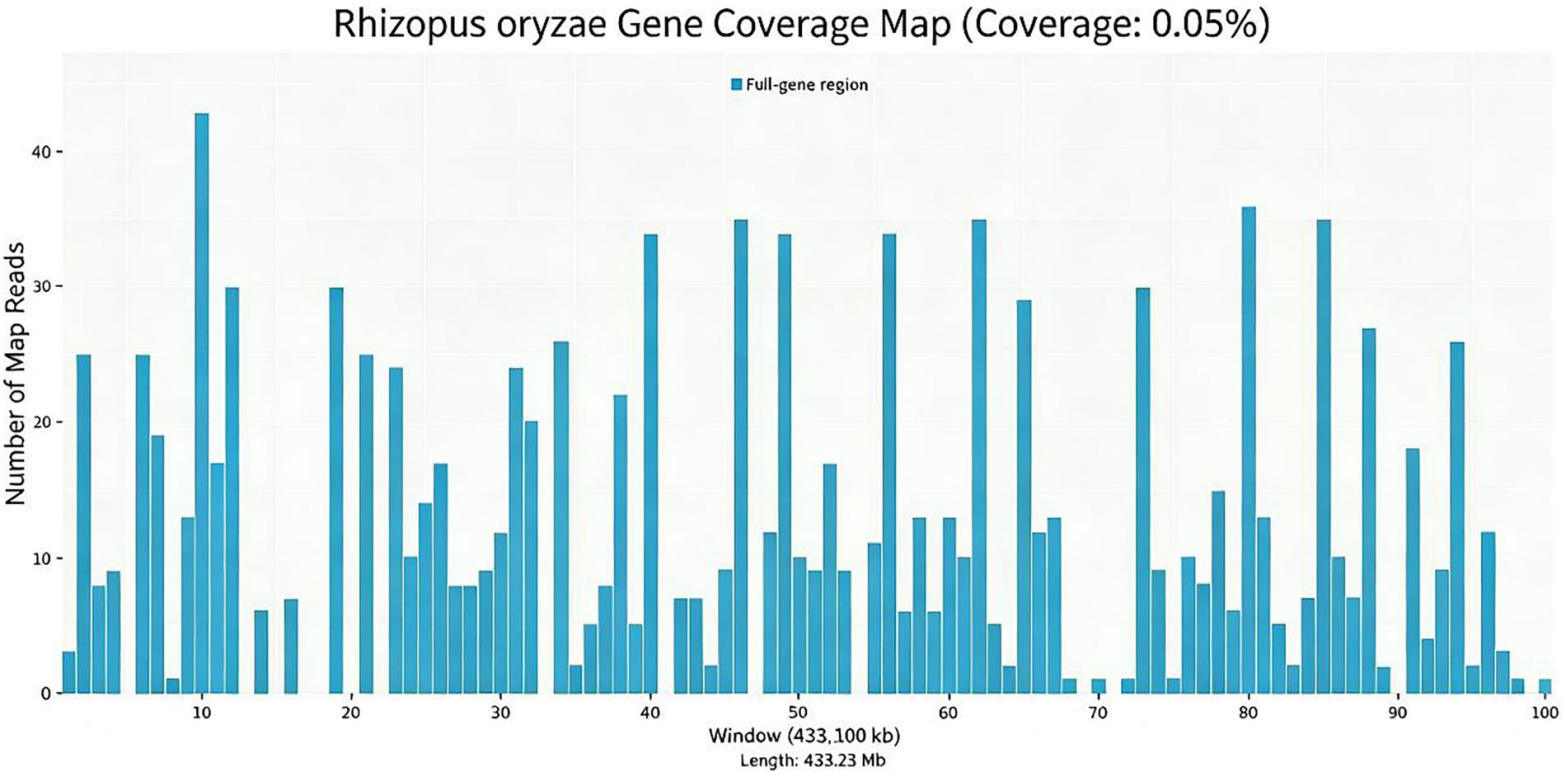
mNGS genome coverage map of *rhizopus oryzae*. mNGS genome coverage map of *Rhizopus oryzae* detected in cerebrospinal fluid. Mapped reads (*n* = 100 windows, 433.03 kb/window) across the 43.3 Mb genome; coverage rate 0.05%. mNGS identified the pathogen 3–5 days earlier than conventional culture, confirming intracranial dissemination.

All 18 surgical patients had histopathological confirmation. HE and GMS staining demonstrated broad, ribbon-like, aseptate hyphae with right-angle branching (Figure 3). Fungal culture was positive in 12/18 (66.7%). PCR-based identification identified Rhizopus spp. in all culture-positive specimens. In 6 patients with intracranial infection, cerebrospinal fluid or abscess cultures yielded Mucorales in all cases. mNGS was performed in 10 (45.5%), with positive detection in 8 (80.0%). Species-level identification by mNGS revealed Rhizopus oryzae in 4 cases and R. arrhizus in 2 cases reducing diagnostic time by 3–5 days compared to conventional culture (Figure 4).

### 3.5 Long-term follow-up clinical outcomes

All patients received standardized 6-month follow-up assessments based on predefined outcome definitions, with extended long-term surveillance conducted for partial survivors beyond the primary 6-month endpoint. Visual function was assessed via Snellen visual acuity chart at baseline, postoperative day 7, as well as postoperative months 1, 3 and 6. Serial sinus CT, cranial MRI and nasal fungal cultures were regularly performed to evaluate lesion clearance, surgical cavity epithelialization and disease recurrence.

In total, 12 patients (54.5%) achieved clinical remission, which required complete resolution of ROCM-associated symptoms, radiological confirmation of total lesion clearance with full mucosal epithelialization of the surgical cavity, and negative nasal fungal cultures. Stratified visual outcomes within the remission cohort were as follows: 6 patients (27.3% of all subjects) obtained meaningful visual recovery with final visual acuity ranging from 0.3 to 0.8, whose baseline visual acuity was merely 0.1 or limited to light perception; 4 patients (18.2% of all subjects) retained stable light perception and recovered full extraocular motility; 2 patients (9.1% of all subjects) showed no improvement in visual acuity but attained complete relief of headache and eyelid edema, meeting all composite remission criteria.

10 patients (45.5%) died within the 6-month primary follow-up period, and all deaths were caused by ROCM-related complications, including progressive intracranial infection (4 patients, 18.2%), multiple organ failure induced by disseminated mucormycosis (4 patients, 18.2%), and fatal postoperative hemorrhage (2 patients, 9.1%). For survivors receiving extended follow-up over 6 months, most maintained disease-free status with well-controlled blood glucose and no recurrent fungal lesions on imaging. 4 patients was lost to follow-up at 12 months after surgery, who showed no sinusitis recurrence and complete surgical cavity epithelialization before loss of contact. 2 patients without ROCM relapse throughout surveillance died of unrelated leukemia at postoperative 6 months and 32 months separately. 6 patients maintained sustained remission during 7–12 months of follow-up, only presenting residual visual defects without disease progression or recurrence.

All 4 non-surgical patients received liposomal amphotericin B; 2 died of progressive intracranial infection, 2 died of refractory multiple organ failure.

### 3.6 Visual acuity outcomes

Among 10 optic nerve decompressed patients, 4 (40.0%) demonstrated meaningful improvement (≥2 Snellen lines); 2 (20.0%) had no light perception preoperatively with no recovery; 4 (40.0%) had stable but poor vision. No patient experienced further visual deterioration post-decompression. Baseline and postoperative visual acuity data are detailed in Table 2.

**Table 2:** Visual acuity outcomes in 10 patients undergoing optic nerve decompression.

Patient	Baseline VA	Post-op Day 7	Post-op Day 30	Post-op Day 180	Outcome
1	HM	0.2	0.4	0.5	Improved
2	LP	0.1	0.3	0.4	Improved
3	0.1	0.2	0.3	0.3	Improved
4	HM	0.15	0.25	0.3	Improved
5	NLP	NLP	NLP	NLP	No change
6	LP	LP	LP	LP	No change
7	0.05	0.05	0.05	0.05	No change
8	HM	HM	HM	HM	No change
9	NLP	NLP	NLP	NLP	No change
10	0.08	0.08	0.08	0.08	No change

VA, visual acuity; HM, hand motion; LP, light perception; NLP, no light perception.

### 3.7 Prognostic univariate and multivariate analyses

**Figure 5:**
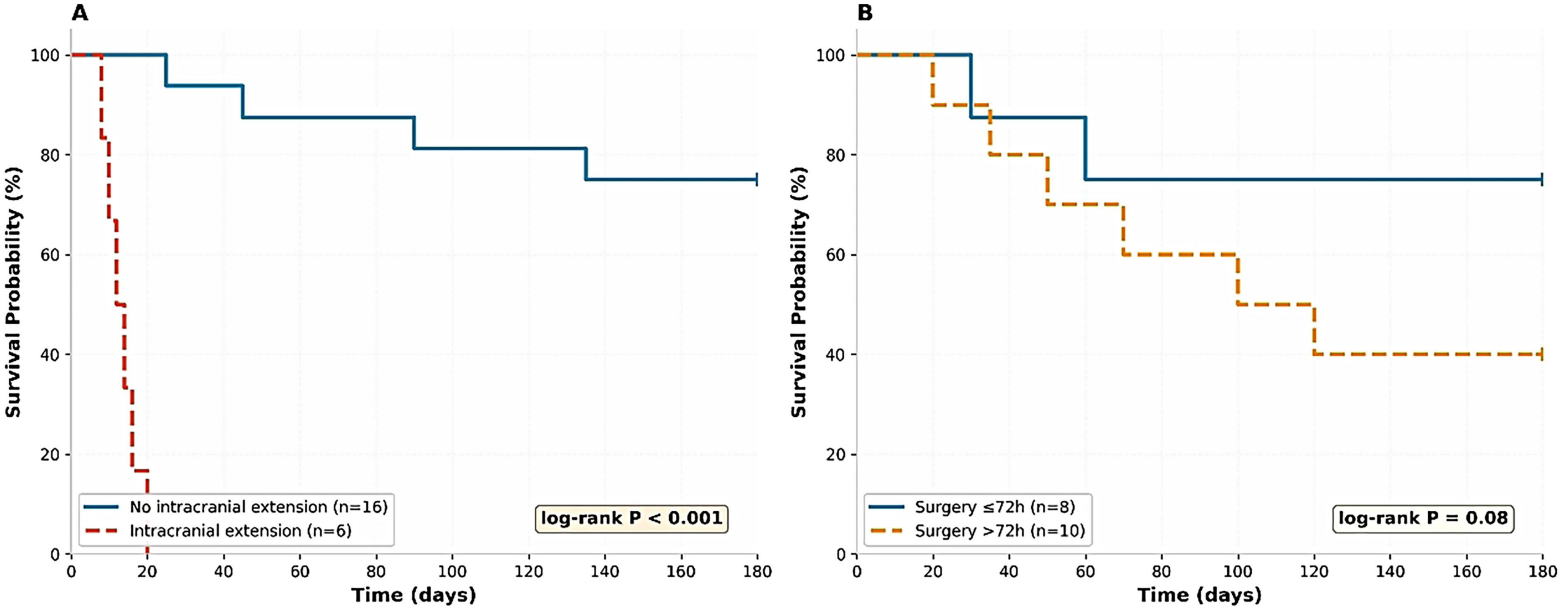
Kaplan–meier survival curves. **(A)** Survival probability by intracranial extension status (log-rank *P* < 0.001). **(B)** Survival probability by surgical timing (log-rank *P* = 0.08).

**Univariate analysis** (Table 3): Intracranial extension (mortality 100% vs 25.0%, *χ*^2^ = 7.35, *P* = 0.007), surgical timing >72 hours (60.0% vs 25.0%, *χ*^2^ = 3.92, *P* = 0.048), and poor diabetes control (66.7% vs 33.3%, *χ*^2^ = 4.28, *P* = 0.039) were significantly associated with mortality.
**Multivariate logistic regression** (Table 4): After adjusting for age, diabetes control, and surgical timing, intracranial extension remained the sole independent predictor of mortality (OR 28.5, 95% CI: 2.1–387.4, *P* = 0.011). Early surgery showed a strong protective trend (OR 0.18, 95% CI: 0.02–1.52, *P* = 0.11) but did not reach statistical significance. Diabetes control also demonstrated a protective trend (OR 0.25, 95% CI: 0.03–2.08, *P* = 0.20).
**Survival analysis** (Figure 5): Kaplan–Meier curves demonstrated significantly superior survival for patients without intracranial extension (median survival not reached vs 14 days, log-rank *P* < 0.001). Patients undergoing surgery ≤72 hours showed improved survival compared to delayed surgery, though the difference did not reach statistical significance (log-rank *P* = 0.08).

**Table 3:** Univariate analysis of prognostic factors for mortality.

Factor	Category	*n*	Deaths	Mortality (%)	*χ* ^2^	*P*-value
Surgical timing	≤72 hours	8	2	25.0	3.92	0.048
	>72 hours	10	6	60.0		
Diabetes control	HbA1c ≤ 7.0%	12	4	33.3	4.28	0.039
	HbA1c > 7.0%	6	4	66.7		
Intracranial extension	Present	6	6	100.0	7.35	0.007
	Absent	16	4	25.0		

Denominator for surgical timing and diabetes control is *n* = 18 (surgical cohort only). Intracranial extension analysis includes all *n* = 22 patients.

**Table 4:** Multivariate logistic regression analysis of mortality predictors.

Variable	OR	95% CI	*P*-value
Age (per 1-year increase)	1.08	0.96–1.22	0.21
Intracranial extension (present)	28.5	2.1–387.4	**0.011**
Surgical timing ≤72 hours	0.18	0.02–1.52	0.11
Diabetes control (HbA1c ≤ 7.0%)	0.25	0.03–2.08	0.2
eGFR ≥60 mL/min/1.73m^2^	0.42	0.05–3.51	0.43

*P < 0.01 (statistically significant by binary logistic regression analysis). OR, odds ratio; CI, confidence interval; eGFR, estimated glomerular filtration rate.

## 4 Discussion

This retrospective cohort study of 22 ROCM patients demonstrates three principal findings: (1) Intracranial extension is the dominant independent predictor of mortality; (2) Early ESS (≤72 hours) and strict glycemic control are strongly associated with improved survival in univariate analysis, though their independent effects require confirmation in larger cohorts; (3)MDT coordination enables optimized surgical timing and comprehensive metabolic management, representing a practical model for ROCM care in tertiary centers.

### 4.1 MDT coordination and diagnostic efficiency

The “symptom dissociation” phenomenon in ROCM contributes to diagnostic delay in over 50% of cases (10). In our cohort, 63.6% of patients initially presented to neurosurgery, consistent with published reports. The MDT protocol reduced time-to-diagnosis by enabling concurrent specialty evaluation: neurosurgical assessment for intracranial complications, endocrinologic optimization of glycemic control, and otorhinolaryngologic evaluation for sinonasal debridement. This approach aligns with Palejwala et al.'s demonstration that aggressive multidisciplinary management reduces mortality in rhinocerebral mucormycosis (11).

The integration of mNGS into our diagnostic workflow reduced pathogen identification time by 3–5 days compared to conventional culture. This acceleration is clinically meaningful in ROCM, where each day of diagnostic delay increases mortality risk (17). However, mNGS cannot replace histopathological examination, as it does not demonstrate tissue invasion—the definitive criterion for invasive fungal disease (4). We advocate for parallel deployment of both modalities in suspected ROCM.

### 4.2 Surgical timing and technical considerations

Our data support the paradigm that early endoscopic debridement is the cornerstone of ROCM management. Among surgical patients, those operated within 72 hours demonstrated a 25% mortality rate versus 60% for delayed surgery. While this difference was not statistically significant in multivariate analysis (*P* = 0.11), the effect size (OR 0.18) suggests clinical relevance that may achieve significance in larger cohorts.

We acknowledge the apparent discrepancy between our 25% early-surgery mortality and the 9.76% figure cited from Sekaran et al. (1). This difference likely reflects: (1) our inclusion of patients with more advanced disease (45.5% with orbital involvement, 27.3% with intracranial extension); (2) longer symptom duration in our cohort (mean 7.2 days vs. undefined in the reference); and (3) potential selection bias toward sicker patients in our tertiary referral center.

Otolaryngologists should focus on the following technical priorities during surgery: **Extent of sinus opening**—the sphenoid and posterior ethmoid sinuses should be opened first, as Mucorales tends to colonize these areas and invade the orbital apex; early opening prevents lesion invasion into the orbital apex and cavernous sinus. **Avoiding over-debridement**—surgery should remove all necrotic tissue, but excessive drilling of the skull base should be avoided (2 patients in this study had cerebrospinal fluid leakage due to over-debridement). Residual devascularized bone can be covered and repaired by mucosa at a later stage (12), reflecting the otolaryngologist's expertise in balancing “functional preservation” with “lesion removal.”

### 4.3 Endoscopic optic nerve decompression

Visual outcomes following optic nerve decompression in ROCM remain variable. In our series, 40% of decompressed patients achieved meaningful visual improvement, while no patient experienced further deterioration. This “stabilization benefit” is consistent with the pathophysiology of ROCM: decompression relieves ischemic pressure on the optic nerve but cannot reverse established axonal necrosis. The window for effective decompression appears narrow; animal models and clinical series suggest maximal benefit when performed within 3–7 days of vision loss (18). Our data support early MDT activation to facilitate rapid surgical decision-making for patients with threatened vision.

### 4.4 Standardized systemic antifungal therapy

Liposomal amphotericin B remains the first-line antifungal for ROCM, with a 20-fold improved therapeutic index compared to conventional amphotericin B (19). In our cohort, 81.8% of patients received liposomal formulation, with 18.2% requiring dose reduction or switch due to nephrotoxicity. The four patients with creatinine elevation >133 μmol/L were successfully managed with flucytosine plus posaconazole, consistent with ECMM salvage recommendations (4).

The combination of amphotericin B plus caspofungin was used in 2 patients with intracranial abscesses, both of whom achieved remission. While Reed et al. reported improved outcomes with combination polyene-caspofungin therapy in a small series (20), we emphasize that our experience is anecdotal and insufficient to support routine adoption. We present this as a hypothesis-generating observation requiring prospective validation.

### 4.5 Prognostic determinants and management algorithm

Our multivariate analysis identifies intracranial extension as the dominant prognostic factor, with 100% mortality in affected patients. This underscores the critical importance of early detection and prevention of intracranial spread through prompt sinonasal debridement. The angioinvasive nature of Mucorales facilitates rapid intracranial extension via the ophthalmic artery, ethmoidal arteries, and cavernous sinus plexus (21). Once intracranial involvement occurs, antifungal penetration is limited by the blood-brain barrier, and surgical access is constrained by critical neurovascular structures. Endoscopic debridement performed by otolaryngologists serves as the radical core therapy to eliminate primary sinonasal lesions, while long-term systemic antifungal treatment acts as an indispensable adjuvant measure to control hematogenous and intracranial dissemination.

Diabetes control and surgical timing, while not independently significant in our multivariate model, demonstrated strong protective trends. Hyperglycemia promotes fungal growth through increased free iron availability and impaired neutrophil chemotaxis (9). Strict glycemic control (HbA1c ≤ 7.0%) may represent a modifiable risk factor amenable to aggressive insulin therapy. The non-significance in multivariate analysis likely reflects limited statistical power; we estimate that a cohort of approximately 80 patients would be required to detect an OR of 0.25 for diabetes control with 80% power.

**Figure 6:**
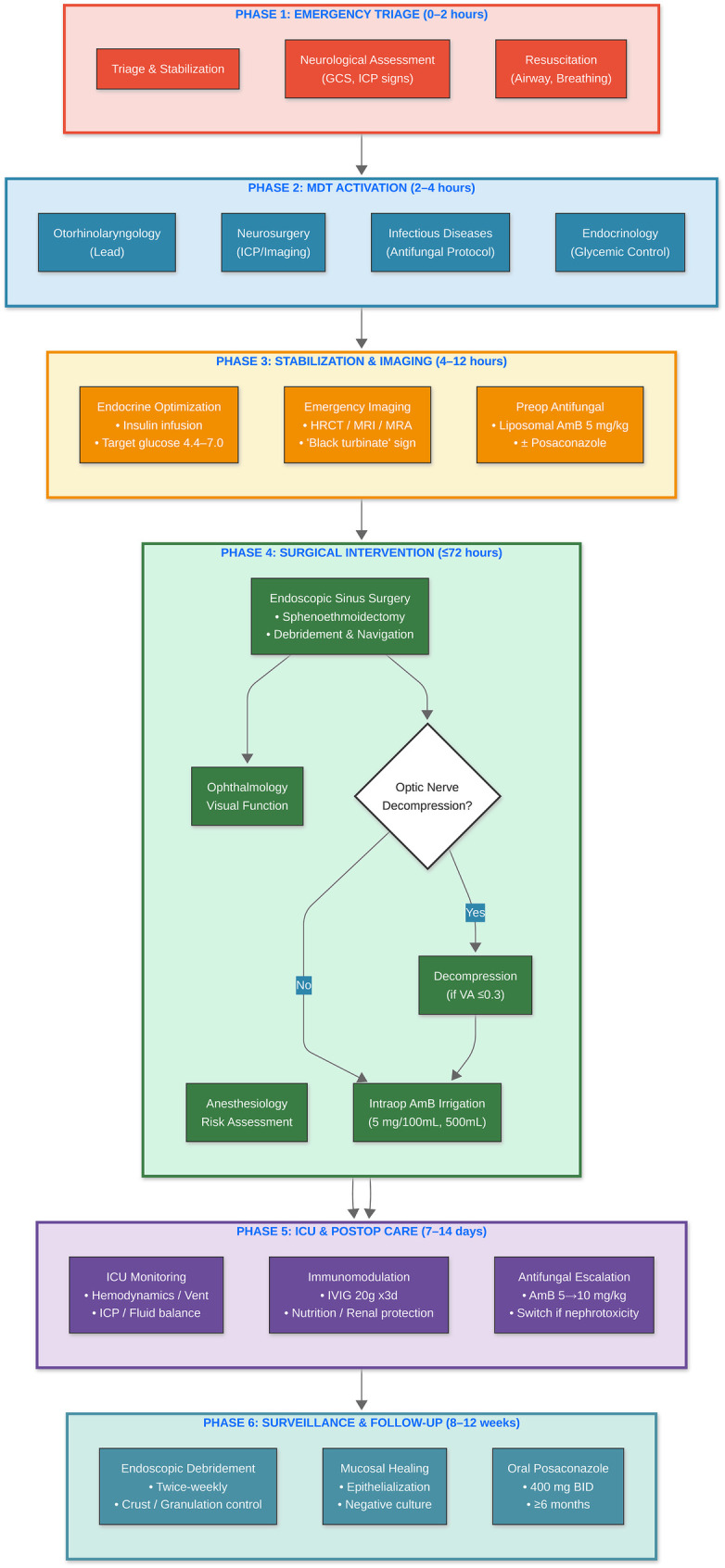
Proposed MDT management algorithm for ROCM. Flow diagram illustrating emergency triage, metabolic stabilization, imaging, MDT conference, surgical intervention, and postoperative surveillance phases.

Based on our experience and published evidence, we propose a standardized MDT algorithm for ROCM management (Figure 6): (1) emergency triage with concurrent endocrinology and infectious diseases consultation; (2) rapid metabolic stabilization (glucose target 4.4–7.0 mmol/L, DKA correction); (3) emergency imaging (CT/MRI) within 2 hours; (4) MDT conference within 4 hours to determine surgical candidacy; (5) endoscopic debridement within 72 hours for all operable patients; (6) intravenous liposomal amphotericin B initiated preoperatively; (7) ICU monitoring with twice-weekly endoscopic surveillance; and (8) step-down to oral posaconazole with long-term follow-up.

### 4.6 Study limitations

This study has several limitations: (1) Retrospective design—data collection relied on medical record review, introducing potential information bias; (2) Small sample size—with 22 patients and 10 mortality events, the study is underpowered to detect moderate effect sizes (minimum detectable OR ≈8.5); (3) Absence of a control group—the 4 non-surgical patients were not prospectively defined controls, and their outcomes cannot be directly compared due to selection bias; (4) Short follow-up—six-month follow-up may underestimate late mortality or recurrence; (5) Selection bias—the high proportion of neurosurgical referrals (63.6%) may reflect severity bias inherent to our tertiary center; (6) Missing data—HbA1c values were not available for all patients at presentation; (7) Statistical constraints—the small sample size precluded reliable multivariate modeling with more than 3–4 predictors.

## 5 Conclusion

Rhino-orbito-cerebral mucormycosis remains a highly lethal infection requiring urgent, coordinated management. This retrospective cohort study of 22 patients demonstrates that intracranial extension is the dominant independent predictor of mortality. Endoscopic sinus surgery within 72 hours of symptom onset, performed within a structured MDT framework with strict glycemic control and optimized antifungal therapy, represents the optimal current management strategy. While early surgery and diabetes control showed strong protective trends, larger prospective studies are required to confirm their independent prognostic value. The proposed MDT algorithm provides a practical framework for tertiary centers managing this devastating disease.

## Data Availability

The raw data supporting the conclusions of this article will be made available by the authors, without undue reservation.
